# Thirdhand cigarette smoke leads to age‐dependent and persistent alterations in the cecal microbiome of mice

**DOI:** 10.1002/mbo3.1198

**Published:** 2021-05-15

**Authors:** Li He, Yan‐Xia Zhou, Yuqing Zhang, Bo Hang, Hang Chang, Suzaynn F. Schick, Susan E. Celniker, Yankai Xia, Antoine M. Snijders, Jian‐Hua Mao

**Affiliations:** ^1^ Biological Systems and Engineering Division Lawrence Berkeley National Laboratory Berkeley CA USA; ^2^ Department of Hematology Zhongnan Hospital Wuhan University Wuhan China; ^3^ Marine College Shandong University Weihai China; ^4^ State Key Laboratory of Reproductive Medicine Institute of Toxicology Nanjing Medical University Nanjing China; ^5^ Department of Medicine Division of Occupational and Environmental Medicine University of California San Francisco CA USA

**Keywords:** 16S rRNA gene sequencing, gut microbiome, mouse exposure, thirdhand cigarette smoke

## Abstract

The gut microbiome composition is influenced by many factors including environmental exposures. Here, we investigated the effect of thirdhand cigarette smoke (THS) and exposure age on gut microbiome diversity. C57BL/6 mice were exposed to THS at human exposure relevant levels for three weeks during three different life stages: postnatal (0–3 weeks of age), pubescent (4–7 weeks of age), and adult (9–12 weeks of age), respectively. Cecal microbiome profiles were assessed at 17 weeks of age by 16S rRNA gene sequencing. We found that age at THS exposure strongly influenced the cecal microbiome composition. Although postnatal THS exposure significantly influenced the microbial composition, pubescent and adulthood exposures only had minor effects. The microbiome of postnatally THS‐exposed mice significantly increased several degradation pathways that regulate glycolysis and pyruvate decarboxylation, and significantly decreased coenzyme A biosynthesis and pyrimidine deoxyribonucleoside salvage. Our results indicate that mouse postnatal development is particularly susceptible to persistent THS exposure effects on the gut microbiome.

## Graphical abstract

Pollutants that are present in dust and remain on surfaces after tobacco has been smoked are collectively called thirdhand smoke (THS). When these pollutants are re‐emitted into the gas phase or react with other environmental chemicals, they have the potential to form secondary pollutants. We investigated the effect of THS exposure during three different life stages (postnatal, pubescent, and adult) on the gut microbiome in mice. Postnatal THS exposure significantly influenced the microbial composition, whereas pubescent and adulthood exposures only had minor effects.

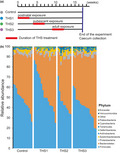

## 1 INTRODUCTION

Significant evidence links the gut microbiome to health and disease, in both human and animal studies (Cryan et al., 2019; Giles & Couper, 2020; Mao et al., 2020; Ogunrinola et al., 2020). The microbiome begins to colonize at birth and becomes established during infancy and early childhood. Environmental factors including diet and xenobiotic exposures can alter the microbiome composition (Leeming et al., 2019; Sun et al., 2020). The adult microbiome is considered relatively robust and can revert backs to its baseline state after environmental exposures (Lozupone et al., 2012). Yet studies show that early‐life environmental exposures have persistent effects on microbiome composition and function (Lozupone et al., 2012; Snijders et al., 2016). In recent years, evidence has emerged that exposure to toxic environmental chemicals leads to adverse health effects that are mediated through altering the gut microbiome (Colquhoun et al., 2020; Khan & Wang, 2019; Sbihi et al., 2019; Tsiaoussis et al., 2019; Tu et al., 2020).

Pollutants that are present in dust and remain on surfaces after tobacco has been smoked are collectively called thirdhand smoke (THS). When these pollutants are re‐emitted into the gas phase or react with other environmental chemicals, they have the potential to form secondary pollutants (Jacob et al., 2017; Matt et al., 2011; Winickoff et al., 2009). The presence of THS has been found widespread in many indoor environments (Matt et al., 2014; Quintana et al., 2013). Unlike its precursor secondhand smoke (SHS), THS toxins can enter the body through different routes including inhalation, ingestion, and skin absorption (Jacob et al., 2017; Matt et al., 2011). Compelling evidence shows that THS causes adverse effects on cells and tissues at environmentally relevant concentrations (Hang et al., 2020). THS exposure causes DNA damage in human and mouse cells (Dhall et al., 2016; Hang et al., 2013, 2018), damage to physiological and psychological processes including effects on body weight, immunity, and behavioral alterations in mice (Hang et al., 2017; Martins‐Green et al., 2014). Low‐dose THS exposure causes specific metabolic changes in mouse male reproductive cells (Xu et al., 2015). A study using the A/J mouse model demonstrated that early‐life exposure to THS increased lung tumor incidence, size, and multiplicity compared to control mice (Hang et al., 2018). The role of the microbiome in mediating THS exposure effects remains unknown.

We investigated the effect of THS exposure and exposure age on the diversity of the gut microbiome in C57BL/6 mice and used bioinformatics analysis to explore alterations in the biological functions associated with the THS‐exposed gut microbiome.

## 2 MATERIALS AND METHODS

### 2.1 THS exposure and cecum collection

**FIGURE 1:**
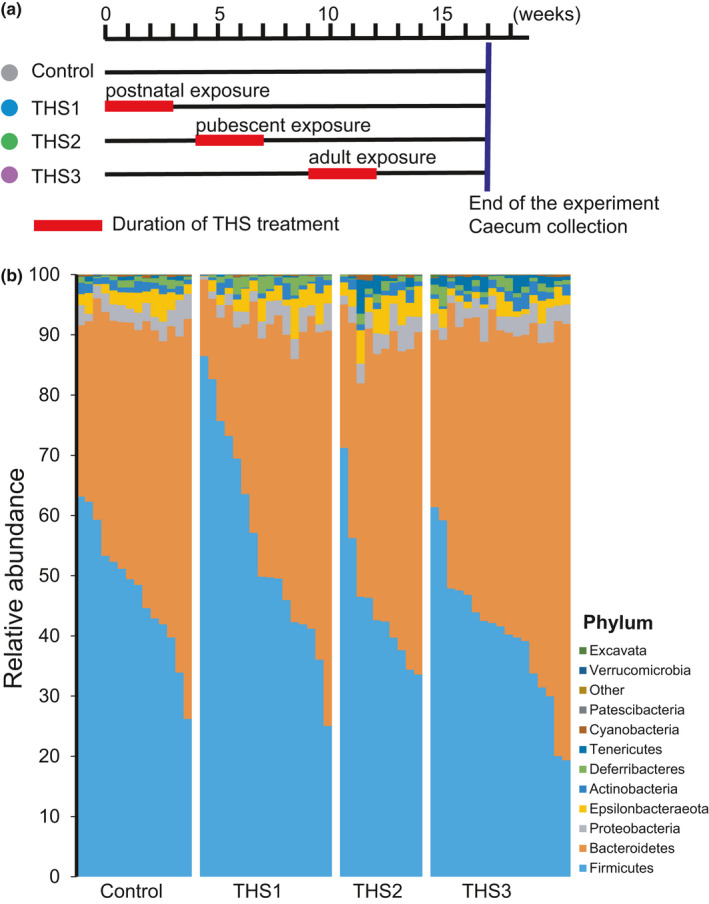
THS exposure alters the relative abundance of the cecal microbiome at the phylum level. (a) Schematic representation of the study design. (b) Distributions of relative abundance at the phylum level in cecal samples from THS‐treated and control mice

The cohorts of C57BL/6 mice were exposed to THS during one of three different life stages: (1) from birth to 3 weeks of age (THS1: postnatal exposure); (2) from 4 to 7 weeks of age (THS2: pubescent exposure); and (3) from 9 to 12 weeks of age (adult exposure) (Figure 1a). The control group was never exposed to THS. For THS1, ten independent litters of mice were randomly selected from our breeding colony. For THS2, THS3, and the control group, mice from independent litters were weaned, separated by sex, and allocated to different cages. Before THS treatment of the THS2 cohort, the cages were randomly assigned into THS2, THS3, or a control group, and there were at least 6 cages per treatment group. At the end of the experiment, one or two mice were randomly selected from each cage for this study. The numbers of mice for each exposure window were: *n* = 14 from 10 cages for control; *n* = 16 for THS1 from 12 cages; *n* = 10 for THS2 from 6 cages; and *n* = 17 for THS3 from 10 cages. All mice were fed a standard chow diet (with a caloric content of 58% carbohydrate, 28.5% protein, and 13.5% fat). THS‐exposed terry cloth was added to the standard bedding in the cages, and the cloth swatches were replaced once a week during the standard cage change. The control group was exposed to same‐sized terry cloth swatches that were not THS‐exposed. The cloth was the sole source of THS exposure. THS compounds in terry cloth substrates were analyzed following the procedures described in our previous study, and the same batch of cloth was used in this study (Hang et al., 2018). All mice were euthanized at 17 weeks of age. Samples of the contents of each cecum were collected on sterile cotton‐tipped swabs, snap‐frozen in liquid nitrogen, and then stored at −80°C.

### 2.2 Microbiome analyses

Genomic DNA was extracted from the cecum samples using the PowerSoil DNA Isolation Kit (http://www.mobio.com/) according to the manufacturer's instructions. PCR amplification of the V4 region of the 16S rRNA gene was performed using the protocol as described in previous studies (Snijders et al., 2016). Amplicons were sequenced on an Illumina MiSeq using paired, 250 base‐pair reads, according to the manufacturer's instructions. The sequence reads were quality‐filtered using QIIME (Quantitative Insights Into Microbial Ecology, V1.9.1). Filtered reads were clustered into operational taxonomic units (OTUs), using an open‐reference picking process with a threshold of 97% similarity to the reference database (Greengenes OTUs (16S) v13_8). The proceeded data from QIIME were provided in Table S1. In addition, we used QIIME to calculate *α* diversity indices, including observed species, Chao1, Shannon, and Simpson. The software package PICRUSt (Phylogenetic Investigation of Communities by Reconstruction of Unobserved States) was used to perform functional analyses.

### 2.3 Statistics analysis

Taxonomic abundance at phylum, family, and genus level was compared using the Mann–Whitney test between THS‐treated and control mice. FDR adjusted *p*‐value (*q*‐value) <0.05 was taken as statistically significant. All analyses and the FDR adjusted *p*‐values were carried out in R (Version 3.6.0). Data were presented in the boxplot with median, interquartile range (IQR), minimum (25th percentile – 1.5*IQR), and maximum (75th percentile + 1.5*IQR). Boxplots were generated using the SPSS statistics package (IBM, Version 24). Principal component analysis and unsupervised clustering analysis were carried based on microbial abundance using R or Clustvis (https://biit.cs.ut.ee/clustvis/) (Metsalu & Vilo, 2015). Specifically, the covariance matrix was constructed based on the normalized abundance of OTUs, and then, the top two components with the largest eigenvalues of the covariance matrix were selected as the principal components as illustrated in the figures. The p‐value for difference between THS1 and other groups was obtained from permutational multivariate analysis of variance (permutational MANOVA, vegan package, version 2.5‐7).

## 3 RESULTS

Three cohorts of C57BL/6 mice were exposed to THS at different life stages: THS1 (postnatal: 0–3 weeks of age), THS2 (pubescent: 4–7 weeks of age), and THS3 (adult: 9–12 weeks of age), and an unexposed cohort of mice served as a control (Figure 1a), which were housed in at least 6 cages per treatment group. At 17 weeks of age, 57 cecum samples were collected for 16S rRNA gene sequencing using Illumina MiSeq (control = 14; THS1 = 16; THS2 = 10; and THS3 = 17). Each sample was rarefied to 54,726 reads that were clustered into 1620 operational taxonomic units (OTUs) at 97% similarity (Table S1).

**FIGURE A1:**
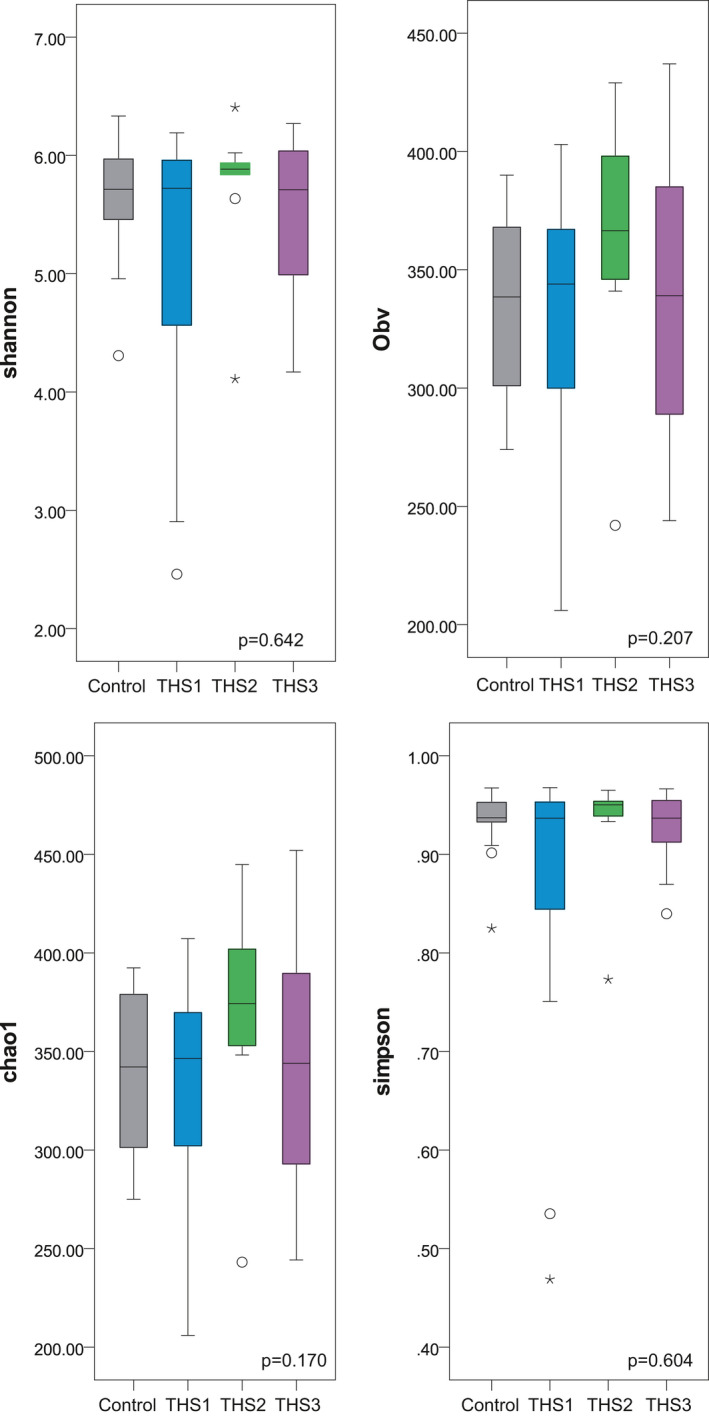
No difference in alpha diversity between THS‐treated and control samples. Alpha diversity based on Chao index, Shannon index, Simpson index, and the number of observed OTUs (Obv) in control, THS1‐, THS2‐, and THS3‐exposed mice. *p*‐values were obtained using the Mann–Whitney test

**FIGURE A2:**
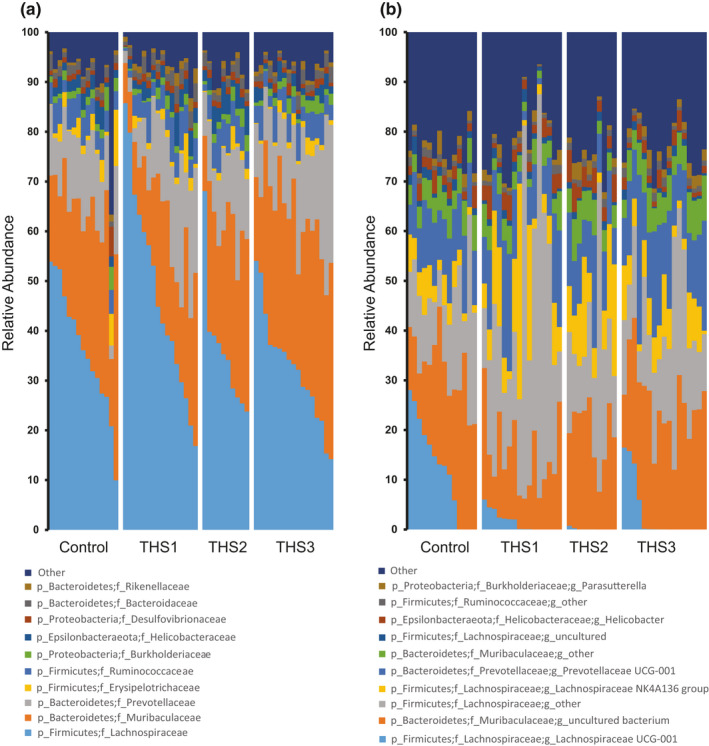
Distributions of abundant families and genera in cecal samples from THS‐treated and control mice. Relative abundance levels of the most common families (a) and genera (b) of control, THS1‐, THS2‐, and THS3‐exposed mice

**FIGURE A3:**
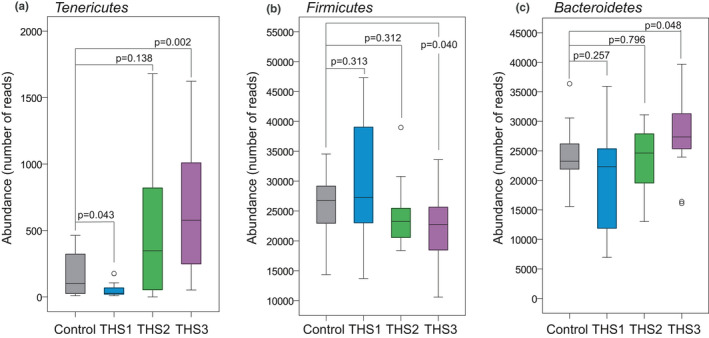
Boxplot of relative abundance of three microbial phyla with different abundance levels between THS‐treated and control groups. Relative abundance levels of Tenericutes (a), Firmicutes (b), and Bacteroidetes (c) in control, THS1‐, THS2‐, and THS3‐exposed mice. *p*‐values were obtained using the Mann–Whitney test

No significant differences were observed in alpha diversity assessed by the number of observed OTUs and Chao, Shannon, and Simpson indices between different treatment groups and the control group (Figure A1 in Appendix 1). However, we observed significant taxonomic differences in the cecal microbiome of THS‐treated and control samples (Figure 1b, Figure A2 in Appendix 1). In comparison to controls, the relative abundances of taxa classified at the phylum level showed significant changes in THS‐treated mice (Figure 1b, Figure A3 in Appendix 1). Postnatal exposure decreased the abundance of the phylum *Tenericutes*, while adult exposure increased it (Figure A3a in Appendix 1). In addition, adult exposure significantly decreased the abundance of the phylum *Firmicutes* (Figure A3b in Appendix 1) and significantly increased the abundance of phylum *Bacteroidetes* (Figure A3c in Appendix 1).

**FIGURE 2:**
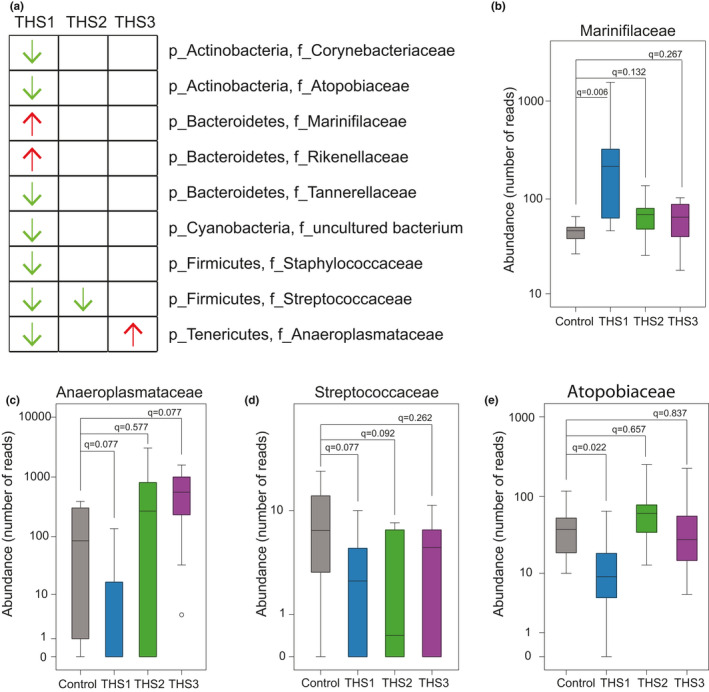
THS exposure alters the relative abundance of the cecal microbiome at the family level. (a) Microbial families significantly altered by THS exposure. Red arrows indicate a significant increase while green arrows indicate a significant decrease in abundance level. (b–e) Boxplots of relative abundance of four representative families showed significant differences between THS‐treated and control animals. The *q*‐value is the FDR adjusted *p*‐value that was obtained from the Mann–Whitney rank test

THS‐induced alterations of the gut microbial composition were also observed at the family and genus levels (Figure A2 in Appendix 1). A total of 59 families were detected in the cecum (Table S2). In comparison to the control group, postnatal exposure significantly altered the abundance levels of nine families. The abundance levels of *Marinifilaceae* and *Rikenellaceae* were significantly increased, whereas seven families including *Anaeroplasmataceae* were significantly decreased (Figure 2a; Mann–Whitney rank test, FDR adjusted *p*‐value *q* ≤ 0.1). Pubescent exposure only significantly decreased the abundance of family *Streptococcaceae* (Mann–Whitney rank test, *q* = 0.092), and adult exposure only significantly increased the abundance of family *Anaeroplasmataceae* (Figure 2, Table S2; Mann–Whitney rank test, *q* = 0.077).

**FIGURE 3:**
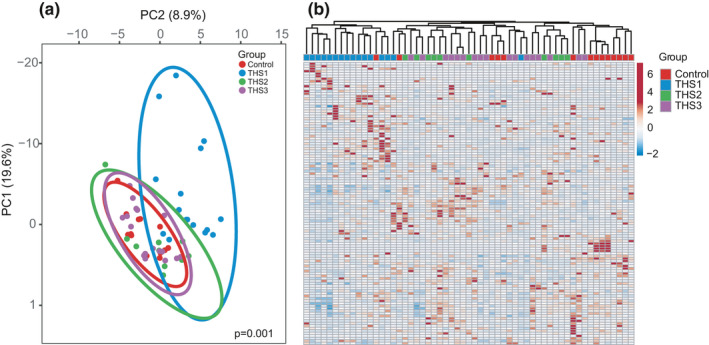
Changes in cecal microbiome composition after THS exposure. (a) Principal component analysis (PCA) of genus‐level microbiomes from THS‐treated and control animals. (b) Hierarchical clustering of the genus‐level microbiome communities from THS‐treated and control animals

**FIGURE 4:**
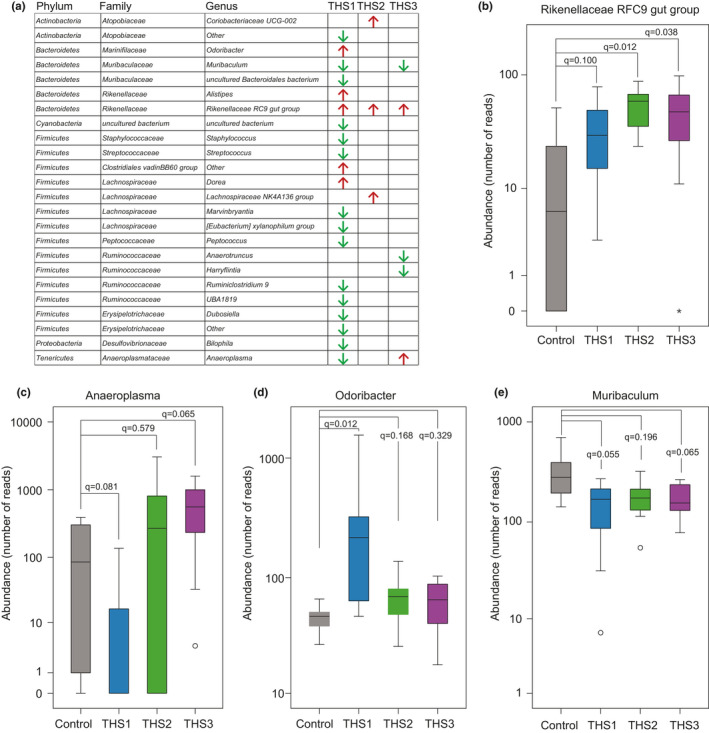
THS exposure alters the relative abundance of the cecal microbiome at the genus level. (a) Genus‐level microbiome communities significantly altered by THS exposure. Red arrows indicate a significant increase while green arrows indicate a significant decrease in abundance. (b–e) Boxplots of the relative abundance of four representative genera showed significant differences between THS‐treated and control animals. The *q*‐value is the FDR adjusted *p*‐value that was obtained from the Mann–Whitney rank test

**FIGURE A4:**
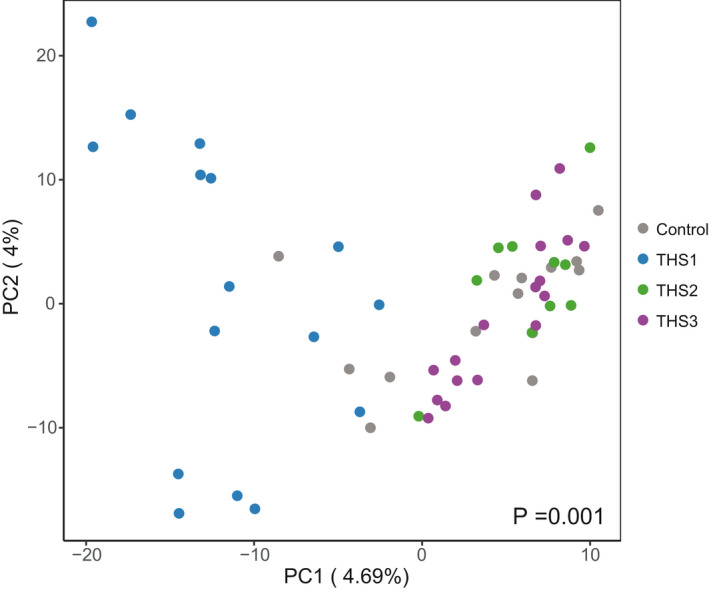
Principal component analysis (PCA) of OTU‐level microbes from THS‐treated and control animals. PCA analysis of the gut microbiome composition at the OTU level in control, THS1‐, THS2‐, and THS3‐exposed mice. *p*‐value was obtained using the Mann–Whitney test

A total of 143 genera were detected, 113 of which have more than 30 sequencing reads in all samples (Table S3). Principal component analysis (PCA) of 113 genera revealed that samples from animals exposed postnatally were significantly different from samples from other life stages (Figure 3a; PERMANOVA *p*= 0.001). A similar observation was obtained by PCA at the OTU level (Figure A4 in Appendix 1; PERMANOVA *p*= 0.001). The unsupervised hierarchical clustering (UHC) segregated THS‐treated samples from controls (Figure 3b). To identify which genera were altered by THS exposure, we compared the relative abundance between postnatal THS‐treated and control samples. We found that the abundance levels of 20 genera differed significantly between the postnatal exposure group and the control group, 15 of which showed a significant decrease in abundance (Figure 4, Table S3; Mann–Whitney rank test, *q* < 0.1). In contrast, the abundance of only three genera was significantly increased between the pubescent exposure group and the control group, and five genera differed significantly in abundance between adult exposure and control group (Figure 4, Table S3; Mann–Whitney rank test, *q* < 0.1). In conclusion, major gut microbiome composition changes were seen in the postnatally THS‐exposed mice, while only minor changes were observed in pubescent and adult exposed mice.

**FIGURE 5:**
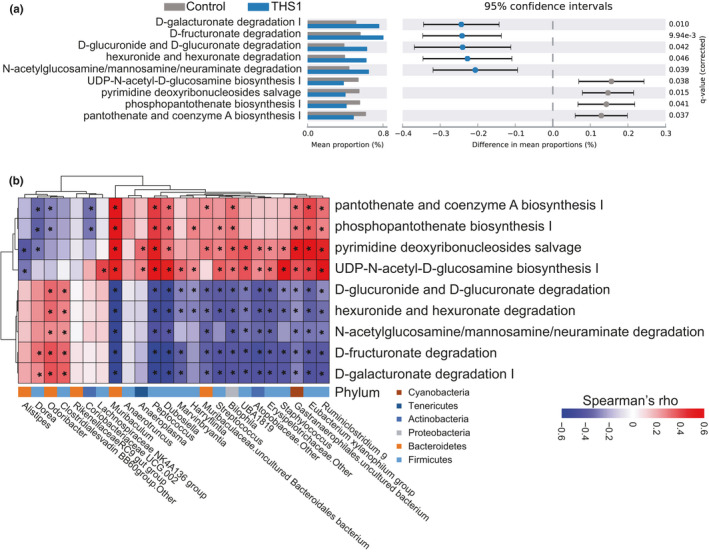
THS exposure alters distinct functional shifts of cecal microbiota. (a) Functional pathways of genera that were significantly altered in mice postnatally exposed to THS using PICRUSt2 analysis. Two‐sided Welch's *t*‐test and FDR correction were used to identify the differentially abundant MetaCyc pathways (*q* < 0.05). (b) Correlations between microbial features at the genera level and MetaCyc pathways. * indicates *p* < 0.05

**FIGURE A5:**
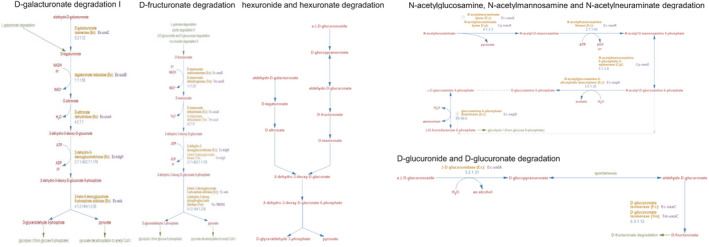
Degradation pathways are altered in the microbiome of postnatally THS‐exposed mice. Biological pathways enriched in the THS‐exposed gut microbiome using PICRUSt2

Finally, we explored the biological functions associated with the THS‐exposed gut microbiome using PICRUSt2. We used this tool to identify biological pathways that might be enriched in the THS‐exposed gut microbiome. Consistent with our observation that pubescent and adult THS exposure had a minor effect on the gut microbiome composition, we did not find any pathway enrichment in the pubescent and adult THS‐exposed microbiomes compared to controls. In contrast, we discovered that the microbiome of postnatally THS‐exposed mice showed a significant increase in several degradation pathways including D‐galacturonate degradation I, D‐fructuronate degradation, D‐glucuronide, and D‐glucuronate degradation, hexuronide, and hexuronate degradation, and N‐acetylglucosamine, N‐acetylmannosamine, and N‐acetylneuraminate degradation, and significantly decreased pyrimidine deoxyribonucleoside salvage and the biosynthesis pathways including UDP‐N‐acetyl‐D‐glucosamine biosynthesis I, phosphopantothenate biosynthesis I, and pantothenate and coenzyme A biosynthesis I (Figure 5a). These degradation pathways are involved in the regulation of glycolysis critical for metabolism and pyruvate decarboxylation the step that links glycolysis to the TCA cycle (Figure A5 in Appendix 1).

To investigate which gut microbes contributed to these pathways, we calculated the correlation between pathway score and genus‐level microbial abundance levels. Significant positive correlations were observed between glucuronoside and galacturonate degradation and abundance of *Dorea*, *Odoribacter*, and the family *Clostridiales* Vadin BB60 group, which are increased in the postnatal THS exposure group (Figure 5b). UDP‐N‐acetyl‐D‐glucosamine biosynthesis and pyrimidine deoxyribonucleoside salvage pathways, which were significantly decreased in microbiomes of postnatally THS‐exposed mice, were positively correlated with a broad range of Firmicutes (Figure 5b).

## 4 DISCUSSION

Nearly, a billion people are current smokers, which contributes to a significant amount of the global health burden (Collaborators, 2017). Smokers’ homes are places where children are primarily exposed to dangerous SHS. These exposures are challenging to regulate by state and federal agencies. In recent years, THS has gained both research and public attention as its widespread presence in the indoor environment as well as potential adverse health impact.

Tobacco‐related diseases, including cardiovascular disease, Crohn's disease, and cancer, are increasing; however, the role or contribution of THS in these diseases remains largely unknown. Environmental exposures are recognized as important factors that can influence the abundance of specific microbes, which subsequently can act as risk factors for chronic diseases (Colquhoun et al., 2020; Tu et al., 2020). Therefore, the adverse health effects of tobacco smoke including THS may be at least in part mediated by modulating the human gut microbiome. To investigate the effect of tobacco smoke on the gut microbiome, we studied the effect of THS exposure during three different life stages on the gut microbiome composition at adulthood. It should be noted that one of the THS exposure routes is ingestion through the GI tract, which may impose a direct impact on the gut microbiome. Previous studies showed that exposure to cigarette smoke altered the gut physiology of rats causing decreased cecal levels of organic acids, elevated pH, and a decrease in the population of Bifidobacterium (Tomoda et al., 2011). A different study in C57BL/6 mice showed that SHS significantly altered the gut microbiome characterized by increased abundance of Clostridium and decreased levels of *Firmicutes*, *Lactococcus*, and *Ruminococcus* (Wang et al., 2012). A human population‐based study showed significant effects of cigarette smoking and the gut microbiome composition (Lee et al., 2018). When compared to never smokers, the gut microbiome of smokers showed increased levels of the phylum Bacteroidetes and decreased levels of *Firmicutes* and *Proteobacteria*. Our study also shows a broad decrease in genera of the phyla Firmicutes and Proteobacteria and increased abundance of several genera of the phylum Bacteroidetes.

It is known that in humans, the gut microbiome establishes in the first three years of life after which the microbiome becomes more adult‐like. In this study, the most sensitive window of THS exposure effects on the microbiome is the postnatal period, right after birth, when the gut microbiome begins to develop and modulate in species abundance. THS exposure stopped at 3 weeks of age, and cecal collections were performed 14 weeks after exposure. These results indicate that early‐life exposure can have persistent effects on the gut microbiome composition. However, it should be noted that the behavior of newborn mice is different from the mice at an older age. Newborn mice within the first 1.5 weeks of life cannot chew THS cloth; therefore, the possible THS exposure routes would be skin absorption and/or inhalation. Transdermal absorption of THS constituents NNA and NNK in laboratory mice has been reported (Jacob et al., 2014). Additionally, it is also noted that two‐phase randomization was used in this study: We first randomly selected the litters for the THS1 cohort, and remaining litters were randomly allocated to different cages during weaning and a week later cages were randomly assigned to control, THS2 or THS3 cohort. Whether different THS exposure routes would influence our observed changes in the gut microbiome warrants further investigation. Nevertheless, our findings are consistent with previous reports that early‐life environmental exposures cause persistent changes in microbiome composition and function, while the adult microbiome is more robust and can revert to its baseline state after exposure (Lozupone et al., 2012; Snijders et al., 2016). Furthermore, a recent comparison of children's microbiomes in THS‐polluted and THS‐free homes showed significant differences in abundance levels of several genera (Kelley et al., 2021). The consequences of THS‐induced early‐life dysbiosis on disease remain to be further investigated.

## 5 CONCLUSION

Increasing evidence has shown a link between adverse health effects and THS exposure, however, the mechanism remains elusive. The gut microbiome provides a new avenue to understand the contribution of THS to the development of disease. We focused on the effect of THS and exposure age on the gut microbiome diversity using a mouse model and the use of bioinformatics analysis to explore alterations in the biological functions associated with the THS‐exposed gut microbiome. Our study shows that THS exposure, especially during early‐life stages, results in significant alterations in the composition of the gut microbiome. This interaction may contribute to the development of disease later in life. Future studies need to address the long‐term health effects or late‐life health effects of THS exposure early in life in relation to alteration in the host gut microbiome.

## CONFLICT OF INTEREST

None declared.

## AUTHOR CONTRIBUTIONS

**Li He:** Formal analysis (supporting). **Yan‐Xia Zhou:** Formal analysis (supporting). **Yuqing Zhang:** Formal analysis (supporting). **Bo Hang:** Formal analysis (supporting); Funding acquisition (equal). **Hang Chang:** Formal analysis (supporting). **Suzaynn F. Schick:** Resources (lead). **Susan E. Celniker:** Formal analysis (supporting); Writing‐review & editing (supporting). **Yankai Xia:** Formal analysis (supporting); Writing‐review & editing (supporting). **Antoine Snijders:** Conceptualization (equal); Formal analysis (equal); Writing‐original draft (supporting). **Jian‐Hua Mao:** Conceptualization (equal); Formal analysis (equal); Funding acquisition (equal); Writing‐original draft (lead).

## ETHICS STATEMENT

All animal experiments were performed at the Lawrence Berkeley National Laboratory, and the study was carried out in strict accordance with the Guide for the Care and Use of Laboratory Animals of the National Institutes of Health. The animal use protocol was approved by the Animal Welfare and Research Committee of the Lawrence Berkeley National Laboratory.

## Supporting information

Table S1

Table S2

Table S3

## Data Availability

The datasets generated and analyzed during the current study are available under NCBI Sequence Read Archive BioProject PRJNA718870: https://www.ncbi.nlm.nih.gov/bioproject/PRJNA718870.
